# Strategies to Improve Antimicrobial Resistant Organism Admission Screening in a Provincial Healthcare System: Consensus-Based Approach

**DOI:** 10.1017/ash.2025.345

**Published:** 2025-09-24

**Authors:** Jenine Leal, Janice Pitchko, Bonita Lee, Logan Armstrong, Philana Choo, Jared Dembicki, Jennifer Ellison, Jayna Holroyd-Leduc, Karen Hope, Jennifer Parsonage, Janine Phipps, Colin Reynolds, Geraldine St Jean, Joseph Vayalumkal, Katelyn Wiley, Samantha Woolsey, John Conly, Elissa Rennert-May, Khara Sauro, Stephanie Smith

**Affiliations:** 1 Alberta Health Services/University of Calgary; 2 Alberta Health Services; 3 University of Alberta; 4 Infection Prevention and Control, Covenant Health; 5 Infection Prevention & Control, Alberta Health Services; 6 University of Calgary; 7 Alberta Childrens Hospital; 8 Foothills Medical Centre

## Abstract

**Background:** Adherence with antimicrobial resistant organism (ARO) admission screening is suboptimal, despite clinical support tools in clinical information systems (CIS) to facilitate the process. Behaviour change techniques to improve adherence are needed. However, in a resource-constrained healthcare system, strategies that motivate healthcare workers (HCWs) to align their practices with infection prevention and control (IPC) policies need to be prioritized. **Methods:** An online survey (REDCap) and a virtual (Zoom) consensus meeting using a modified nominal group technique with online voting was conducted among HCWs, IPC, and the CIS staff in September and October 2024, respectively, to achieve consensus on a prioritized list of interventions to improve ARO admission screening at acute care and acute rehabilitation facilities (n=100) in Alberta, Canada. Interventions from the Behaviour Change Wheel were mapped to barriers/enablers influencing screening adherence. Each intervention was judged across the APEASE criteria (Acceptability, Practicality, Effectiveness, Affordability, Side Effects, Equity) using a 5-point Likert Scale. Consensus to include interventions required >4 criteria with >80% agreement, consensus to exclude required >4 criteria with 80%. Interventions that did not reach consensus were discussed to determine whether to include in the final candidate list. Attendees were asked to vote on their top three interventions from the final candidate list. **Results:** There were 15 barriers and one enabler to ARO admission screening, mapped to 43 unique interventions. Of these, 16 interventions addressed more than one barrier/enabler, while 27 interventions only addressed a single barrier. Fifty-nine respondents completed the survey. Most respondents (63%) were IPC staff, 20% were nurses, and 17% were other HCWs (including IPC physicians). Nine interventions met criteria to include in the candidate list, 26 were excluded, and 8 interventions did not reach consensus in the survey and were discussed. There were 32 attendees at the consensus meeting (53% IPC staff and physicians, 34% clinical staff, 13% other provincial teams). Three interventions were selected: 1) creating a nursing task to complete the tool in the CIS when an admission order is signed, 2) add a banner on the CIS Storyboard when the tool is not complete, and 3) develop a best practice guideline for frontline staff on ARO admission screening. **Conclusions:** The survey and consensus meeting were efficient methods to determine a prioritized list of interventions, which will be implemented and evaluated, to improve ARO admission screening in Alberta.

